# Safeguarding Well‐Being Under Exploitative Leaders: The Buffering Effects of Follower Strategies on Perceived Injustice

**DOI:** 10.1002/smi.70127

**Published:** 2025-11-28

**Authors:** Saleh Bajaba, Saad Basaad

**Affiliations:** ^1^ King Abdulaziz University Jeddah Saudi Arabia; ^2^ University of Business and Technology Jeddah Saudi Arabia

**Keywords:** conservation of resources, employee workplace well‐being, exploitative leadership, interactional injustice, job demands‐resources, managing your boss

## Abstract

This study examines the impact of exploitative leadership (EL) on employees' workplace well‐being (WWB), elucidating underlying mechanisms and mitigating factors. Grounded in Conservation of Resources (COR) theory, the Job Demands‐Resources (JD‐R) model, and the Transactional Theory of Stress (TTS), we explore the mediating role of interactional injustice (IIJ) in the EL‐WWB relationship and the moderating effect of managing‐your‐boss (MYB) strategies. Using a two‐wave time‐lagged design, data were collected from 263 full‐time U.S. employees and analysed via Partial Least Squares Structural Equation Modeling (PLS‐SEM), supplemented by Importance‐Performance Map Analysis (IPMA) and Necessary Condition Analysis (NCA). Results indicate that EL positively relates to IIJ, which in turn negatively affects WWB, establishing IIJ as a full mediator. MYB moderates the EL‐IIJ link, weakening it at higher MYB levels, and extends this buffering to the conditional indirect pathway. IPMA underscores IIJ's high negative importance for WWB, while NCA reveals no necessary conditions but highlights MYB's enabling role. These findings advance destructive leadership research by emphasising follower agency in resource conservation and stress appraisal, offering practical insights for enhancing well‐being through proactive strategies. Theoretical implications, limitations, and future research directions are discussed.

## 1 Introduction

In an era where low employee engagement costs the global economy 9.6 trillion dollars in lost productivity annually, exacerbating burnout, turnover, and mental health challenges amid rising stress (40% of workers) and declining well‐being (Gallup 2025), understanding the darker side of leadership is more critical than ever. Destructive leadership behaviors, such as abusive supervision and exploitation, directly fuel these crises: meta‐analytic evidence reveals they account for up to 30%–40% of variance in employee emotional exhaustion, turnover intentions, and health impairments, including increased absenteeism and psychological distress (Montano et al. 2017; Schyns and Schilling 2013). For instance, in healthcare settings, toxic leadership has been linked to 40% higher burnout rates and elevated turnover, underscoring its role in amplifying organizational costs and human suffering (Labrague et al. 2020; Harms et al. 2017).

Building on this broad evidence, the leadership literature has increasingly spotlighted destructive styles that harm subordinates and organizations by prioritising self‐interest over collective goals (Mackey et al. 2017; Tepper 2000; Schmid et al. 2018; Schyns and Schilling 2013). Among these, exploitative leadership (EL) has emerged as a particularly insidious form, eroding employee commitment, disrupting work–life balance, and provoking psychological distress (Alajhar et al. 2024; Diebig and Bormann 2020; Lyu et al. 2023; Schmid et al. 2019). Cross‐cultural evidence further links EL to diminished justice perceptions and heightened emotional turmoil (Vogel et al. 2015). Recent studies extend these insights by showing that EL fosters unethical conduct, knowledge withholding, reduced employee passion, and declines in well‐being (S. Bajaba et al. 2025; Basaad et al. 2023; Khalid and Aftab 2024; Hussain et al. 2024; Krasikova et al. 2013; Nie and Wang 2025; Pircher Verdorfer et al. 2024). Despite this growing evidence, most research has focused on outcomes and individual coping traits, leaving unanswered how followers might proactively counteract exploitation through strategic behaviors.

Against this backdrop, what remains unclear is the precise mechanism by which leaders' self‐interested exploitation depletes followers' resources and the extent to which employees can proactively counteract these effects (S. Bajaba et al. 2022). Prior studies have largely examined the direct outcomes of EL, such as reduced well‐being and ethical silence, while giving little attention to proactive follower responses like strategic upward management (e.g., A. Bajaba et al. 2023; Schmid et al. 2019; Wang et al. 2024). Moreover, research has often overlooked the relational processes through which resource depletion unfolds, particularly perceptions of injustice that erode trust and equity, focussing instead on individual traits or organizational factors. For example, Elahi et al. (2025) demonstrated that exploitative leadership undermines subjective well‐being among software professionals in Pakistan through personal traits such as self‐efficacy and resilience, yet this work neglected relational dynamics like interactional injustice (IIJ) and proactive strategies such as managing‐your‐boss (MYB), which may operate across diverse occupational roles. This gap is important because it reflects a continued emphasis on passive buffers (e.g., resilience) rather than active follower agency in reframing and mitigating destructive dynamics.

Exploitative leadership is defined as “leadership with the primary intention to further the leader's self‐interest by exploiting others” across dimensions including egoism, credit taking, pressure exertion, development undermining, and manipulation (Schmid et al. 2019, 1404). It represents a subtle yet insidious form of destructive leadership. Unlike overtly hostile styles such as abusive supervision, which involves direct aggression and humiliation (Tepper 2000), EL's manipulative tactics often produce longer‐term harm by covertly eroding interpersonal trust and perceptions of equity (Schmid et al. 2019). Leaders influence employee attitudes and outcomes through resource dynamics (Dai and Xie 2016), where exploitative acts can trigger perceptions of IIJ, defined as unfair treatment that violates dignity and informational clarity (Colquitt 2001; Colquitt et al. 2005), ultimately impairing workplace well‐being (WWB) (Skogstad et al. 2017).

To address these theoretical and practical gaps, this study integrates, **c**onservation of resources (COR) theory, the job demands resources (JD‐R) model, and the transactional theory of stress (TTS) into a cohesive framework. COR posits that individuals strive to protect and acquire resources, with threats like EL leading to stress spirals and negative outcomes (Hobfoll 1989; Hobfoll et al. 2018). JD‐R complements this by framing EL as a resource depleting job demand that hinders well‐being unless offset by buffers (Bakker and Demerouti 2017). TTS adds a coping lens, viewing stress as an appraisal process where proactive responses can reframe threats (Lazarus and Folkman 1984). Together, these theories explain how EL fosters IIJ as a mediator in eroding WWB, while follower strategies like MYB, proactive alignment with supervisors (Gajendran et al. 2022), may moderate this pathway by conserving resources and enabling adaptive reappraisal (Krasikova et al. 2013; Martinko et al. 2011).

**FIGURE 1:**
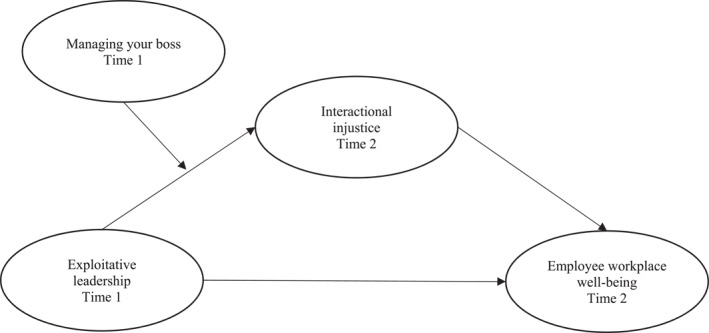
The conceptual model.

**FIGURE 2:**
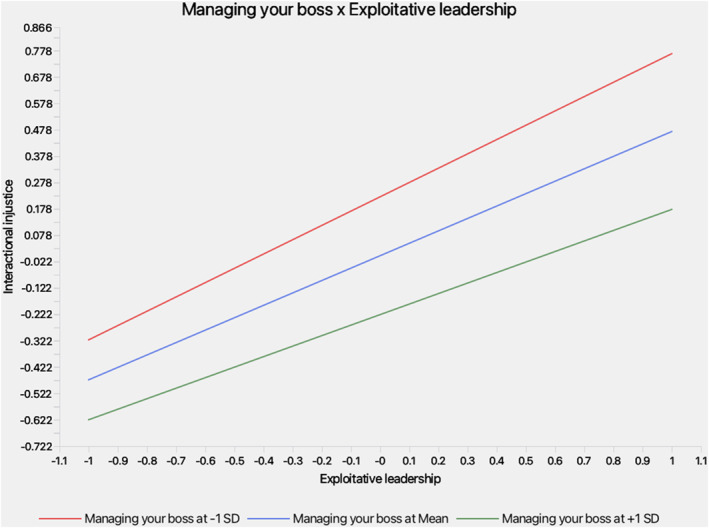
Interacting effects of exploitative leadership with managing your boss on interactional injustice.

This study makes theoretical, methodological, and practical contributions to the literature on destructive leadership and workplace well‐being. Theoretically, it integrates COR theory, the JD‐R model, and the TTS to model how follower agency counters EL, extending the EL–WWB nomological network by positioning IIJ as a mediating mechanism and MYB as a proactive moderator (Schmid et al. 2019; Gajendran et al. 2022). Methodologically, it advances analytical rigor through a two‐wave time‐lagged design with 263 U.S. employees and by employing Partial Least Squares Structural Equation Modeling (PLS‐SEM) for model testing, Importance‐Performance Map Analysis (IPMA) for predictor prioritisation (Ringle and Sarstedt 2016), and Necessary Condition Analysis (NCA) for identifying threshold effects (Dul 2016; Sarstedt et al. 2024). This multimethod approach addresses common challenges in moderation and mediation research, such as insufficient power and overlooked necessary conditions (Aguinis et al. 2017; Dawson 2014). Practically, it offers organizations actionable strategies, including implementing MYB training programs and monitoring IIJ perceptions, to mitigate EL's detrimental effects and strengthen employee resilience. Collectively, these contributions deepen theoretical understanding, strengthen methodological precision, and provide evidence‐based practices to combat toxic leadership (see Figure 1, Figure 2).

### 1.1 Theoretical Background

Exploitative leadership (EL), as defined by Schmid et al. (2019), involves leaders prioritising their self‐interests through behaviors such as genuine egoism, taking undue credit, exerting excessive pressure, undermining follower development, and manipulation. These actions are often triggered by perceived obstacles to goal attainment, including contextual factors like resource scarcity (e.g., budget, time, information) and dispositional traits such as narcissism Machiavellianism (Krasikova et al. 2013; Schmid et al. 2019; S. Bajaba et al. 2022). For instance, exploitative leaders may claim credit for subordinates' achievements to gain recognition or impose additional burdens on followers to advance personal agendas, disregarding the harm inflicted (Schmid et al. 2019; S. Bajaba et al. 2022). Emerging research highlights EL's distinctiveness from other destructive styles, showing links to outcomes like psychological distress, unethical behavior, and reduced well‐being (Alajhar et al. 2024; S. Bajaba et al. 2025; Basaad et al. 2023; Khalid and Aftab 2024; Hussain et al. 2024; Nie and Wang 2025; Pircher Verdorfer et al. 2024). To further differentiate EL, Mackey et al. (2017)'s meta‐analysis on abusive supervision reveals that while abusive supervision involves overt hostility and direct aggression, EL's sneaky manipulation, such as covert credit‐taking and subtle undermining, inflicts more insidious harm, often evading immediate detection and leading to prolonged resource erosion (Schmid et al. 2019). However, gaps persist in understanding the perceptual mechanisms (e.g., injustice) and follower‐driven buffers that mitigate EL's resource‐depleting effects.

COR theory provides a foundational lens, positing that individuals are driven to obtain, retain, protect, and build resources, defined as anything valued for survival or goal achievement (Hobfoll 1989; Hobfoll et al. 2018). Key tenets include resource loss primacy (losses outweigh gains in impact), investment for protection (proactive resource building to prevent future losses), and loss spirals (initial losses accelerate further depletion). In this context, EL acts as a chronic stressor that erodes followers' personal resources (e.g., energy, self‐esteem, autonomy) and job resources (e.g., fairness, support), leading to stress, emotional exhaustion, and diminished WWB (Halbesleben et al. 2014). For example, manipulative tactics like credit taking threaten relational resources, fostering perceptions of interactional injustice (IIJ) and amplifying negative outcomes.

Complementing COR, the JD‐R model frames work conditions as demands (energy‐draining aspects) or resources (motivational aids; Demerouti et al. 2001; Bakker and Demerouti 2017; Bakker et al. 2004). Central tenets distinguish health impairment processes (high demands like EL deplete resources, causing burnout) from motivational processes (resources promote engagement). EL represents an emotional and cognitive demand that overwhelms interpersonal resources, heightening IIJ perceptions and undermining WWB. However, personal resources such as proactive behaviors can buffer these effects, preserving motivation and health. To examine coping, the TTS serves as a supportive framework, conceptualising stress as a dynamic person‐environment transaction involving primary appraisal (assessing threats) and secondary appraisal (evaluating coping resources; Lazarus and Folkman 1984). TTS tenets emphasise adaptive coping strategies, distinguishing problem‐focused (directly addressing stressors) from emotion‐focused (managing reactions). Followers may appraise EL as a threat to dignity, prompting proactive strategies like managing your boss (MYB) to reframe demands, conserve resources, and reduce IIJ, thereby enhancing well‐being.

Integrating these theories creates a framework for understanding EL's impacts (Shepherd and Suddaby 2017); COR elucidates resource depletion mechanisms, JD‐R highlights demand‐resource imbalances and buffering, and TTS explains appraisal and coping processes. Together, they extend destructive leadership research by modeling how EL indirectly erodes WWB via IIJ, moderated by MYB, offering novel propositions that address calls for follower‐centric, multi‐theory approaches (Krasikova et al. 2013). To fully elucidate these processes, we detail the mediating and moderating mechanisms below.

The mediation of IIJ in the EL‐WWB relationship stems from the relational resource depletion driven by EL's manipulative behaviors. According to COR's resource loss principle, tactics such as credit‐taking and pressure exertion erode interpersonal resources, leading to IIJ, perceptions of unfair treatment that violate dignity and clarity (Schmid et al. 2019; Hobfoll et al. 2018). This is reinforced by JD‐R's health impairment process, where EL's chronic demands heighten IIJ, subsequently impairing WWB (Bakker and Demerouti 2017). This relational focus addresses a gap in prior research, such as Elahi et al. (2025), which emphasised personal traits like self‐efficacy. For moderation, MYB acts as a proactive buffer, enabling followers to conserve resources and reappraise stressors. TTS's coping framework suggests that MYB facilitates alignment with supervisors, reducing IIJ's impact through adaptive reappraisal, a process supported by empirical evidence of proactive strategies lowering stress (Lazarus and Folkman 1984; Gajendran et al. 2022). This highlights a follower‐driven approach, building on the oversight of proactive strategies in earlier studies. These mechanisms lay the groundwork for testing their effects in subsequent analyses.

## 2 Hypothesis Development

### 2.1 Exploitative Leadership and Interactional Injustice

Interactional justice refers to the quality of interactions that subordinates experience with their immediate superiors or other individuals holding authority (Bies and Moag 1986). This concept is multifaceted, encompassing factors like the perception of being treated with politeness, dignity, and respect by authoritative figures. It also includes evaluating whether these figures have provided appropriate justifications for decisions or outcomes (Colquitt 2001). Interactional justice bifurcates into two distinct subcategories: interpersonal justice, concerning the treatment received by employees, and informational justice, which pertains to the clarity of explanations or justifications given about work procedures and outcomes (Colquitt 2001).

From a COR perspective, EL depletes followers' socioemotional resources, such as trust and respect, by prioritising the leaders gains at the expense of subordinates (Hobfoll et al. 2018). This resource loss manifests as perceived IIJ, where manipulative behaviors (e.g., taking credit or exerting pressure) erode relational resources (Halbesleben et al. 2014). In JD‐R terms, EL acts as a chronic job demand that overwhelms available resources, heightening strain and fostering injustice perceptions (Bakker and Demerouti 2017). TTS further explains this as a primary appraisal process, where followers evaluate EL as a threat to dignity, leading to negative reappraisals of fairness (Lazarus and Folkman 1984). Recent research by Pircher Verdorfer et al. (2024) reinforces this by distinguishing EL's subtle manipulation from abusive supervision, showing how it uniquely erodes trust and triggers IIJ. Additionally, Alajhar et al. (2024) and Hussain et al. (2024) provide evidence that EL's manipulative tactics directly correlate with heightened injustice perceptions across contexts. Empirical evidence supports that destructive leadership, including exploitative forms, correlates with heightened injustice perceptions, reduced prosocial behavior, and diminished well‐being (Wu et al. 2021; Ye et al. 2022; Syed et al. 2021; Sun et al. 2022; S. Bajaba et al. 2022), as well as deviant responses under power imbalances (Tepper et al. 2009). Therefore, we hypothesise.

Hypothesis 1 Exploitative leadership is positively related to interactional injustice.

### 2.2 Interactional Injustice and Employee Workplace Well‐Being

The relationship between organizational justice and employee well‐being has gained significant attention in recent decades, with evidence suggesting that justice perceptions are critical to employee health and well‐being (Colquitt 2001; Fujishiro and Heaney 2009). The absence of justice, particularly interactional justice, has been linked to increased job stress, health complaints, absenteeism, and negative physiological outcomes, such as cardiovascular and immunological issues (Elovainio et al. 2001; Elovainio et al. 2003; Kivimäki et al. 2003; De Boer et al. 2002; Heponiemi et al. 2011). Interpersonal justice, in particular, shapes employees' stress perceptions, profoundly influencing their overall well‐being (Judge and Colquitt 2004; Moliner et al. 2008).

Workplace interpersonal conflicts, such as those arising from EL, are emotionally and physically demanding, impacting employees' ability to maintain professional relationships and achieve organizational objectives (M. T. M. Dijkstra et al. 2011; Demerouti et al. 2001; Peeters et al. 2005). WWB, encompassing job satisfaction and positive feelings at work, is undermined by such conflicts, which correlate with depressive symptoms, burnout, and somatic complaints (Page and Vella‐Brodrick 2009; Slemp et al. 2015; Dormann and Zapf 1999; Hacer and Ali 2020; Frone 2000). IIJ amplifies job dissatisfaction, turnover intention, and negative emotions, such as frustration and anger, further eroding WWB (Ambrose and Schminke 2009; Hagemeister and Volmer 2018; Spector and Jex 1998).

From a COR perspective, IIJ represents a loss of relational resources, exacerbating the stress caused by EL and leading to a decline in WWB (Hobfoll et al. 2018). JD‐R frames IIJ as a consequence of high demands (EL) that deplete resources, impairing health and motivation (Bakker and Demerouti 2017). TTS complements this by suggesting that employees appraise IIJ as a stressor, reducing their ability to cope with workplace demands (Lazarus and Folkman 1984). Thus, we hypothesise.

Hypothesis 2 Interactional injustice is negatively related to employee workplace well‐being.

### 2.3 The Mediating Role of Interactional Injustice

Building on the direct association outlined above, we now integrate COR, JD‐R, and TTS to propose that IIJ mediates the relationship between EL and WWB. EL depletes resources, fostering perceptions of unfair treatment (IIJ), which in turn erodes WWB through increased stress, dissatisfaction, and absence (Hobfoll et al. 2018; Bakker and Demerouti 2017; Bakker et al. 2003). Complementing this resource‐based perspective, social exchange theory (SET; Cropanzano et al. 2017) highlights how EL disrupts normative reciprocity in leader‐follower relationships, manifesting as perceived IIJ that further impairs WWB through imbalanced exchanges. TTS supports this by framing IIJ as a secondary appraisal outcome, where demands are seen as uncontrollable threats (Lazarus and Folkman 1984). Recent work by Wang et al. (2024) highlights how EL, through work meaningfulness and moral potency, fosters ethical silence, amplifying its negative impact on employee outcomes and supporting this mediating role. This mediation aligns with prior research showing that justice perceptions mediate the effects of destructive leadership on well‐being outcomes (e.g., Syed et al. 2021; Pircher Verdorfer et al. 2024). Empirical studies confirm that exploitative leadership indirectly affects well‐being through self‐efficacy (Elahi et al. 2025). Therefore, we hypothesise.

Hypothesis 3 Interactional injustice will mediate the relationship between exploitative leadership and employee workplace well‐being.

### 2.4 The Moderating Role of Managing Your Boss

The Job Demands Resources (JD‐R) model provides insight into how workplace demands and resources influence employee well‐being and performance (Demerouti et al. 2001; Bakker and Demerouti 2017). EL represents a high job demand that depletes resources, but personal resources, such as proactive behaviors, can buffer these effects by fostering engagement and reducing strain (Bakker and Demerouti 2007; Xanthopoulou et al. 2009). TTS complements this by emphasising coping strategies, such as problem‐focused coping (e.g., managing your boss), which help manage demands through adaptation (Lazarus and Folkman 1984; Catalano et al. 2011).

Managing your boss (MYB) involves employees aligning with their supervisors' goals and communication styles to enhance job performance and leader member exchanges (Gajendran et al. 2022; Graen and Scandura 1987). MYB is a proactive coping strategy that allows employees to conserve resources by aligning with the leaders' priorities, reducing the perceived threat of EL (Kwon and Kim 2019). From a COR perspective, MYB mitigates resource loss by fostering control and relational resources, while JD‐R views it as a personal resource that buffers the negative effects of job demands (Bakker and Demerouti 2017; Hobfoll et al. 2018). TTS suggests that MYB enables employees to reappraise EL as a manageable challenge, reducing IIJ perceptions (Lazarus and Folkman 1984). However, we recognise potential downsides: in highly toxic environments, ramping up MYB could exacerbate resource depletion, leading to increased strain, as shown in Strauss et al. (2017) on the hidden costs of proactivity when motivation is low. Despite this, MYB focuses specifically on IIJ, rather than distributive or procedural justice, because it targets interpersonal and informational dynamics with supervisors, directly countering EL's relational violations.

Empirical evidence supports that proactive behaviors, like MYB, enhance employee efficacy in navigating challenging leadership contexts (Gajendran et al. 2022; Sherf et al. 2019). Lyu et al. (2023) show exploitative leadership evokes deviance via moral justification under high hostile attribution bias, harming well‐being; we extend this by proposing positive agency (e.g., MYB) as a mitigator. By aligning with the leaders' goals, employees can mitigate the injustice perceptions arising from EL, thereby weakening the EL‐IIJ relationship. Thus, we hypothesise.

Hypothesis 4 Managing your boss will moderate the relationship between exploitative leadership and interactional injustice, such that the relationship is weaker when managing your boss is high (vs. low).

#### 2.4.1 The Moderated Mediation Model

Building on the established mediation and moderation, we propose MYB moderates the indirect effect of EL on WWB via IIJ. In COR terms, MYB interrupts resource loss spirals by protecting relational resources, weakening the mediated pathway (Hobfoll et al. 2018). The JD‐R posits that personal resources like MYB attenuate demand strain links, particularly in high demand contexts (Bakker and Demerouti 2017). TTS frames this as adaptive coping that alters appraisals, reducing the conditional impact of injustice on well‐being (Lazarus and Folkman 1984). Recent research by Nie and Wang (2025) suggests that ego depletion under EL can be mitigated by proactive behaviors, supporting MYB's moderating role. This aligns with JD‐R's buffering hypothesis, where personal resources reduce demand‐strain effects (Bakker and Demerouti 2017).

Empirical support for moderated mediation in similar models (see, Tepper et al. 2017) suggests MYBs buffering extends to indirect effects. However, as Strauss et al. (2017) note, proactivity's costs, such as added strain in low‐motivation scenarios—could limit MYB's effectiveness in extremely toxic settings, though its focus on IIJ's relational aspects makes it particularly suited for moderating EL's interpersonal harms over other justice forms. Thus, we hypothesise.

Hypothesis 5 Managing your boss will moderate the negative indirect relationship of exploitative leadership with employee workplace well‐being via interactional injustice, such that the negative indirect relationship is weaker when managing your boss is high (vs. low).

## 3 Materials and Methods

### 3.1 Data Collection Procedures and Sample

We employed a two‐wave design to temporally separate predictors from outcomes, consistent with recommendations for testing process models in leadership research (Antonakis et al. 2010). All participants provided informed consent, and procedures complied with institutional ethical standards as well as the 1964 Helsinki Declaration and its later amendments. At Time 1 (T1), participants evaluated EL and MYB. Two weeks later, at Time 2 (T2), they assessed IIJ and WWB.

The 2‐week interval was chosen based on Dormann and Griffin (2015), who showed that short lags are effective for capturing acute perceptual shifts in stress‐related dynamics, such as those triggered by EL, while also supporting participant retention. We acknowledge, however, that this duration may underestimate longer‐term well‐being effects, which often require extended lags; we highlight this issue in our future research recommendations. Importantly, a short lag reduces priming effects and supports causal interpretation (Podsakoff et al. 2012), since 2 weeks is generally sufficient to detect perceptual changes in leadership and well‐being dynamics, particularly in contexts of ongoing stress appraisal (Gabriel et al. 2019).

Although longer lags could further reinforce temporal precedence, our design balanced methodological rigor with practical constraints, helping to reduce common method variance without eliminating all risks of reverse causality (e.g., baseline well‐being shaping leadership perceptions). We addressed this concern by including relevant controls and acknowledging it as a study limitation.

The survey was distributed via Prolific, a high‐quality crowdsourcing platform for behavioral research (Douglas et al. 2023; A. Newman et al. 2021; Peer et al. 2022), targeting full time U.S. employees in public and private sectors. Despite recent questions about online panel quality, Prolific consistently yields reliable data compared to alternatives like MTurk, with lower rates of inattention and bots (Peer et al. 2022; Douglas et al. 2023). Participants received compensation via Prolific, aligned with the platform's guidelines of at least 8.00 dollars per hour (recommended 12.00 dollars per hour), amounting to approximately 1.50–2.00 dollars per survey wave based on estimated completion times. Participants with less than 6 months tenure with their current leader were screened out to ensure sufficient exposure to leadership behaviors (Schmid et al. 2019; S. Bajaba et al. 2022; A. Bajaba et al. 2023). An initial 274 responses were collected; after listwise deletion of 11 invalid cases (failed attention checks), the final sample was 263 (D. A. Newman 2014). This sample size meets guidelines for PLS SEM (minimum 100 to 200 cases; Hair et al. 2021) and provides adequate power (ratio of 15 observations per variable; Hair et al. 2019), though we note potential underpowering for complex moderated mediation, addressed via bootstrapping.

The convenience sample via Prolific may introduce biases, such as overrepresentation of certain demographics (60% male and a mid‐career skew, with 45% aged 30–39 years), which could influence perceptions of injustice and coping (e.g., varying by tenure or gender). This demographic imbalance is noted as a limitation that may affect generalisability. However, Prolific quality controls and U.S. focus enhance representativeness for full time employees across sectors, though generalisability is limited to similar contexts. Demographics: 60% male, 40% female; mean age category: 30–39 years (range: 19 to greater than 60; frequencies: 19 to 29: 16%, 30 to 39: 45%, 40 to 49: 23%, 50 to 59: 15%, greater than 60: 1%); mean tenure with current leader: over 18 months and less than 3 years (range: greater than 6 months to greater than 5 years); mean organizational tenure: 2–5 years (range: 7–12 months to greater than 5 years); mean overall experience: over 18 months and less than 3 years (range: greater than 6 months to greater than 5 years). See Table 1 for details.

**TABLE 1:** Sample characteristics.

Variables	Frequency (*N* = 263)	Percentage (%)
Age		
19 to 29	42	16
30 to 39	118	45
40 to 49	60	23
50 to 59	40	15
60 and more	3	1
Gender		
Male	159	60
Female	104	40
Education		
High school diploma/A‐levels	44	16.7
Technical/community college	36	13.7
Undergraduate degree (BA/BSc/other)	121	46
Graduate degree (MA/MSc/MPhil/other)	46	17.5
Doctorate (PhD/other)	16	6.1
Organizational tenure		
7–12 months	9	3.4
1–2 years	43	16.3
2–5 years	78	29.7
More than 5 years	133	50.6
Work experience with current leader		
Less than 6 months	0	0
More than 6 months and less than 18 months	65	24.7
Over 18 months and less than 3 years	58	22.1
3 years and less than 5 years	62	23.6
Over 5 years	78	29.7
Work experience overall		
Less than 6 months	0	0
More than 6 months and less than 18 months	6	2.3
Over 18 months and less than 3 years	10	3.8
3 years and less than 5 years	11	4.2
Over 5 years	236	89.7

### 3.2 Measures and Scales

All constructs were measured using validated scales with five‐point Likert‐type responses (1 = strongly disagree to 5 = strongly agree), unless noted. Reliability exceeded thresholds (Cronbach's α > 0.70; composite reliability > 0.70). *Exploitative Leadership* (EL): 15‐item scale by Schmid et al. (2019); sample: “My leader takes it for granted that my work can be used for his or her personal benefit” (*α* = 0.95). *Interactional Injustice* (IIJ): nine‐item scale adapted from Niehoff and Moorman (1993), originally developed to measure interactional justice based on Moorman (1991). To assess injustice rather than justice, all items were reverse scored (e.g., high scores indicate greater perceived injustice). No items were inherently reverse coded in the original scale. Sample: ‘When decisions are made about my job, the general manager treats me with kindness and consideration’ (reverse‐scored; α = 0.96). Reverse scoring is common in justice research to capture injustice directly, though it may introduce response biases; we mitigated this via CFA accounting for method effects and confirmed discriminant validity.

*Managing‐Your‐Boss* (MYB): 10‐item scale adapted from Gajendran et al. (2022); sample: “I proactively attempt to get a better understanding of my boss's priorities” (*α* = 0.91). *Workplace Well‐Being* (WWB): six‐item scale by Zheng et al. (2015); sample: “I am satisfied with my work responsibilities” (*α* = 0.92). Control variables, age, gender, education, tenure with the current leader, and overall experience, were included and dummy‐coded or treated as categorical where appropriate. See supplementary material for all scale items.

To mitigate potential common method bias (CMB), we implemented several procedural remedies and conducted statistical tests. Procedurally, we ensured anonymity, counterbalanced item order, and included a theoretically unrelated marker variable, attitudes towards the color blue, measured with a seven‐item scale (Miller and Simmering 2022; Lindell and Whitney 2001). Statistically, we performed Harman's single‐factor test, which explained 37% of the variance, falling below the 50% threshold and indicating no substantial CMB (Podsakoff et al. 2003). This result is supported by our time‐lagged design and HTMT ratios (< 0.85), which further confirm the data's robustness against CMB (Henseler et al. 2015). Additionally, partial correlation analysis, using the marker variable, showed no significant CMB influence, though the use of single‐source self‐reports may increase shared variance risks, suggesting multi‐source validation as a future research direction.

### 3.3 Data Analysis

Descriptive statistics and correlations were computed using SPSS 29. Structural relationships were tested via partial least squares structural equation modeling (PLS SEM) in SmartPLS 4.0 (Ringle et al. 2020; Sarstedt et al. 2022), suitable for predictive models, complex paths (e.g., moderated mediation), and emerging theories like EL processes (Hair et al. 2021; Latan 2018; Hair et al. 2022). PLS SEM was chosen over covariance‐based SEM (CB SEM) for its robustness to non‐normality, smaller samples, and focus on prediction rather than strict model fit, which aligns with our exploratory moderated mediation (Henseler et al. 2016). While CB SEM could assess global fit for confirmatory purposes, PLS SEM better handles our reflective constructs and interaction terms without distribution assumptions.

Significance levels were determined using bootstrapping with 5000 subsamples and bias‐corrected accelerated confidence intervals (Hair et al. 2021). To extend the sufficiency‐oriented focus of PLS‐SEM, we incorporated Importance‐Performance Map Analysis (IPMA) to prioritise predictors (Ringle and Sarstedt 2016) and Necessary Condition Analysis (NCA) to identify critical bottlenecks (Dul 2016; Richter et al. 2020). This integrative approach addresses both sufficiency and necessity logics, thereby enhancing the robustness and depth of the insights generated (Hauff et al. 2024; Sarstedt et al. 2024).

## 4 Results

Data analysis was conducted using SmartPLS 4.0 (Ringle et al. 2020) to evaluate the structural relationships in the proposed model (Figure 1), aligning with the resource‐based (COR, JD‐R) and stress appraisal (TTS) frameworks that emphasise dynamic processes of depletion, buffering, and coping. The PLS algorithm was employed with default settings (path weighting scheme, 300 iterations maximum; Latan 2018). Significance of path coefficients, t‐statistics, and *p*‐values was assessed via bootstrapping with 5000 subsamples and no sign changes, reporting bias‐corrected and accelerated (BCa) 95% confidence intervals (Hair et al. 2021). This approach supports causal inferences from the time‐lagged design, testing how EL depletes resources (via IIJ) and how MYB buffers these effects on WWB.

The PLS‐SEM analysis followed a two‐stage process: measurement model evaluation for reliability and validity, followed by structural model assessment for hypotheses (Hair et al. 2021). Multiple indicators enabled simultaneous testing of relationships, consistent with the model's mediated and moderated paths (Hayes 2018; Preacher et al. 2007).

### 4.1 Measurement Model

The measurement model was assessed per Hair et al. (2021) guidelines to ensure construct reliability and validity.

#### 4.1.1 Reliability

As shown in Table 2, factor loadings ranged from 0.61 to 0.90, exceeding the 0.50 threshold for item reliability (Hair et al. 2019; Latan 2018). All loadings were significant (*t* ≥ 1.96, two‐tailed, *p* < 0.05; Roldán and Sánchez‐Franco 2012). Cronbach's alpha (CA) and composite reliability (rho_a) values exceeded 0.80 for all constructs, confirming internal consistency (Hair et al. 2021).

**TABLE 2:** Measurement model results.

Constructs	Items	FL	VIF	CA	Rho_a	AVE	Square root of AVE
Exploitative leadership	EL1	0.83	3.31	0.95	0.96	0.61	0.78
	EL2	0.82	3.29				
	EL3	0.73	2.23				
	EL4	0.78	2.88				
	EL5	0.83	4.40				
	EL6	0.78	3.27				
	EL7	0.78	3.36				
	EL8	0.86	2.07				
	EL9	0.76	3.29				
	EL10	0.81	3.09				
	EL11	0.77	2.82				
	EL12	0.84	3.49				
	EL13	0.71	2.32				
	EL14	0.76	4.26				
	EL15	0.76	4.42				
Interactional injustice	IIJ1	0.86	3.46	0.96	0.96	0.74	0.86
	IIJ2	0.85	3.37				
	IIJ3	0.87	3.64				
	IIJ4	0.85	3.14				
	IIJ5	0.87	3.66				
	IIJ6	0.82	2.63				
	IIJ7	0.90	4.42				
	IIJ8	0.90	4.35				
	IIJ9	0.87	3.29				
Managing your boss	MYB1	0.72	1.98	0.91	0.94	0.55	0.74
	MYB2	0.62	1.99				
	MYB3	0.75	2.85				
	MYB4	0.82	3.07				
	MYB5	0.82	2.77				
	MYB6	0.81	2.22				
	MYB7	0.73	2.72				
	MYB8	0.75	1.69				
	MYB9	0.61	1.94				
	MYB10	0.76	1.53				
Employee workplace well‐being	WWB1	0.78	2.34	0.92	0.93	0.72	0.85
	WWB2	0.90	3.97				
	WWB3	0.88	4.07				
	WWB4	0.78	2.35				
	WWB5	0.84	3.41				
	WWB6	0.88	3.09				

Abbreviations: AVE = Average variance extracted. Squared AVE = Square root of Average variance extracted; CA Cronbach's Alpha; CR (rho_a) = Composite reliability; EL = Exploitative leadership; FL = Factor loadings; IIJ = Interactional injustice; MYB = Managing your boss; VIF = Variance inflation factor; WWB = Employee workplace well‐being.

#### 4.1.2 Validity

Convergent validity was supported by average variance extracted (AVE) values > 0.50, indicating constructs explained at least 50% of indicator variance (Hair et al. 2021). Discriminant validity was confirmed via the Fornell‐Larcker criterion (square root of AVE > inter‐construct correlations) and heterotrait‐monotrait (HTMT) ratios < 0.85 (Table 3; Fornell and Larcker 1981; Franke and Sarstedt 2019; Henseler et al. 2015).

**TABLE 3:** Discriminant validity results from Fornell‐Larcker, HTMT, and Correlations.

Variables	EL	IIJ	MYB	WWB
EL	**0.78**	0.58^h^	0.22^h^	0.34^h^
IIJ	0.56	**0.86**	0.36^h^	0.67^h^
MYB	−0.22	−0.37	**0.74**	0.34^h^
WWB	−0.34	−0.64	0.33	**0.85**
EL	—	−0.55**^b^	−0.18*^b^	−0.31**^b^
IIJ	0.56**^c^	—	−0.32**^b^	−0.63**^b^
MYB	−0.19**^c^	−0.33**^c^	—	0.30**^b^
WWB	−0.32**^c^	−0.63**^c^	0.30**^c^	—
Mean	2.23	3.77	2.23	3.67
Standard deviation	0.90	0.72	0.90	0.90

*Note:* *N* = 263, *|t| ≥ 1.65 at *p* 0.05 level; **|t| ≥ 2.33 at *p* 0.01 level; ***|t| ≥ 3.09 at *p* 0.001 level.

Abbreviations: EL = Exploitative leadership; IIJ = Interactional injustice; MYB = Managing your boss; WWB = Employee workplace well‐being; Below the diagonal are the values of the Fornell‐Larcker. Above the diagonal are the values of the h = HTMT values (heterotrait‐monotrait ratio); b = correlations controlled by the marker variable (attitude towards blue color); c = raw correlations.

#### 4.1.3 Model Fit

The standardized root mean square residual (SRMR = 0.071) fell below 0.08, suggesting a good fit (Hu and Bentler 1999; Henseler et al. 2016). Exact fit metrics showed minimal deviation (d_ULS = 4.085 vs. saturated 3.975; d_*G* = 1.660 vs. saturated 1.651), and chi‐square was efficient (estimated 2138.099 < saturated 2147.182; T. K. Dijkstra and Henseler 2015). The normed fit index (NFI = 0.80) was acceptable for predictive PLS‐SEM models prioritising variance explanation over absolute fit (Bentler and Bonett 1980; Hair et al. 2021).

### 4.2 Correlation Analysis

Table 3 presents correlations. EL positively correlated with IIJ (*r* = 0.56, *p* < 0.01) and negatively with WWB (*r* = −0.34, *p* < 0.01), aligning with resource depletion expectations (Hobfoll et al. 2018). The high correlation between EL and IIJ is theoretically expected, since EL's manipulative behaviors often foster perceptions of unfair treatment (Schmid et al. 2019; Pircher Verdorfer et al. 2024). However, the constructs remain conceptually distinct: EL emphasises leader intent, whereas IIJ captures follower perceptions of interactional fairness (Colquitt 2001). Discriminant validity is further demonstrated through HTMT ratios well below the 0.85 threshold (0.58; Henseler et al. 2015), acceptable VIF values (< 5), and evidence of unique variance in the model, such as IIJ's independent mediation effect, confirming the absence of redundancy. IIJ negatively correlated with WWB (*r* = −0.64, *p* < 0.01), supporting stress appraisal links (Lazarus and Folkman 1984). MYB negatively correlated with EL (*r* = −0.22, *p* < 0.01) and IIJ (*r* = −0.37, *p* < 0.01) but positively with WWB (*r* = 0.33, *p* < 0.01), indicating buffering potential (Bakker and Demerouti 2017). Marker variable controls confirmed no CMB influence (Lindell and Whitney 2001).

## 5 Structural Model

Variance inflation factors (VIFs) ranged from 1.53 to 4.42 (all < 5.00 threshold), ruling out multicollinearity and CMB (Kock 2015; Hair et al. 2021). The model explained 39% of the variance in IIJ (*R* ^2^ = 0.39, Adj *R* ^2^ = 0.38) and 42% in WWB (*R* ^2^ = 0.42, Adj *R* ^2^ = 0.40), indicating moderate explanatory power (Hair et al. 2021). Effect sizes (f^2^) were medium for IIJ predictors (0.32) and large for WWB (0.49; Cohen 2013). Predictive relevance was adequate (*Q* ^2^ = 0.34 for IIJ, 0.14 for WWB > 0), with the smaller Q^2^ for WWB still indicating positive relevance for this model's outcomes (Fornell and Cha 1993).

A post‐hoc power analysis using G*Power (Faul et al. 2009) indicated that with a sample size of 263, *α* = 0.05, and five predictors, the minimum detectable effect size at 80% power was *f* ^2^ ≈ 0.031. In our model, the observed effect sizes were notably larger, with *f* ^2^ = 0.32 for interactional injustice and *f* ^2^ = 0.49 for workplace well‐being (Table 4). These correspond to medium‐to‐large effects according to Cohen's (2013) benchmarks, suggesting that our study was well powered (> 0.99) to detect the observed effects, exceeding conventional adequacy thresholds (Faul et al. 2009; Hair et al. 2021).

**TABLE 4:** Structural model results.

	Interactional injustice				Employee workplace well‐being			
	*Q* ^2^ *=* 0.34, *R* ^2^ = 0.39, Adj*R* ^2^ = 0.38, *f* ^2^ *=* 0.32				*Q* ^2^ = 0.14, *R* ^2^ = 0.42, Adj*R* ^2^ = 0.40, *f* ^2^ *=* 0.49			
Variables	**Path coefficients**	**95% Bca confidence interval**	**T Statistics**	*p* **Value**	**Path coefficients**	**95% Bca confidence interval**	**T Statistics**	*p* **Value**
Control variables								
AGE	0.00	(−0.10, 0.11)	0.03	0.98	0.13	(0.03, 0.24)	2.49	0.01
GEN	0.01	(−0.09, 0.11)	0.10	0.92	0.03	(−0.07, 0.14)	0.66	0.51
EDU	−0.04	(−0.13, 0.06)	0.77	0.44	−0.02	(−0.11, 0.07)	0.44	0.66
WexpL	−0.05	(−0.15, 0.05)	1.04	0.30	−0.02	(−0.12, 0.08)	0.33	0.74
WexpO	−0.06	(−0.18, 0.06)	0.93	0.35	−0.08	(−0.17, 0.01)	1.71	0.09
Direct effect								
EL	0.47	(0.37,0.56)	9.57	0.01	0.02	(−0.09, 0.14)	0.40	0.69
IIJ					−0.65	(−0.75, −0.53)	12.08	0.01
MYB	−0.22	(−0.33, −0.14)	4.50	0.01				
EL*MYB	−0.07	(−0.15, −0.00)	1.98	0.04				

Variables		Path coefficients	95% Bca confidence interval		T Statistics			*p* Value
Specific indirect effect								
EL − > IIJ − > WWB		−0.31	(−0.40, −0.23)		7.13			0.01
Conditional direct effect								
MYB x EL − > IIJ + 1 SD		0.40	(0.27, 0.52)		6.24			0.01
MYB x EL − > IIJ mean		0.47	(0.37, 0.56)		9.57			0.01
MYB x EL − > IIJ −1 SD		0.54	(0.42, 0.65)		9.33			0.01
Conditional indirect effect								
MYB x EL − > IIJ − > WWB + 1 SD		−0.26	(−0.36, −0.17)		5.33			0.01
MYB x EL − > IIJ − > WWB mean		−0.31	(−0.39, −0.22)		7.13			0.01
MYB x EL − > IIJ − > WWB − 1 SD		−0.35	(−0.45, −0.26)		7.12			0.01

*Note:* |*t*| ≥ 1.65 at *p* 0.05 level; |*t*| ≥ 2.33 at *p* 0.01 level; *|t*| ≥ 3.09 at *p* 0.001 level.

Abbreviations: BCa = Bias‐corrected and accelerated. *R* ^2^ = Determination coefficients; *Q* ^2^ = Predictive relevance of endogenous; EL = Exploitative leadership, IIJ = Interactional injustice, MYB = Managing your boss, WWB = Employee workplace well‐being; AGE = Age; EDU = Education; GEN = Gender; WexpL = Work experience with current leader; WexpO = Work experience overall.

### 5.1 Hypothesis Testing

Bootstrapping results (Table 4) supported all hypotheses. H1: EL positively affected IIJ (*β* = 0.47, *t* = 9.57, *p* < 0.001, 95% BCa CI [0.37, 0.56]), consistent with resource threat perceptions. H2: IIJ negatively affected WWB (*β* = −0.65, *t* = 12.08, *p* < 0.001, 95% BCa CI [‐0.75, −0.54]), reflecting depletion outcomes. H3: IIJ fully mediated EL‐WWB (indirect *β* = −0.31, *t* = 7.13, *p* < 0.001, 95% BCa CI [‐0.40, −0.23]; direct non‐significant). H4: MYB moderated EL‐IIJ (interaction *β* = −0.07, *t* = 1.98, *p* = 0.047, 95% BCa CI [‐0.15, −0.00]); stronger at low MYB (‐1SD: *β* = 0.54) versus high (+1SD: *β* = 0.40). While statistically significant at *p* < 0.05, this effect is small in magnitude (Cohen 2013), as is typical for interactions in this domain (Aguinis et al. 2017; Dawson 2014), indicating context‐dependent buffering by MYB that merits further validation in larger or varied samples (Hair et al. 2021). H5: Moderated mediation index positive (*β* = 0.05, *t* = 1.96, *p* = 0.050, 95% BCa CI [0.00, 0.09]); indirect weaker at high MYB (+1SD: *β* = −0.26) versus low (‐1SD: *β* = −0.35).

### 5.2 Importance‐Performance Map Analysis

**FIGURE 3:**
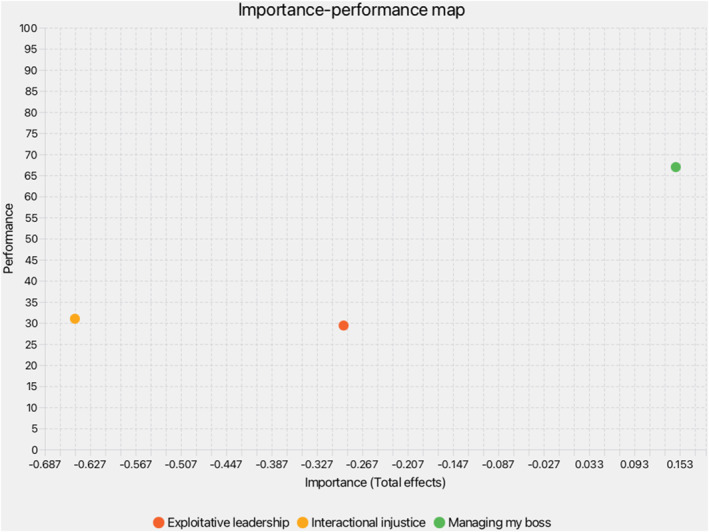
The graphical representation of the importance‐performance map analysis.

IPMA (Figure 3, Table 5) extended PLS‐SEM by prioritising predictors (Ringle and Sarstedt 2016). MYB showed high performance (66.96) but moderate importance (0.15) for WWB. IIJ had medium performance (31.00) but high negative importance (−0.65), emphasising injustice reduction. EL had low performance (29.40) and negative importance (−0.29), aligning with depletion effects (Hobfoll et al. 2018).

**TABLE 5:** Index values and total effects of importance‐performance map analysis.

Variables	Total effect on employee workplace well‐being (importance)	Index values (performance)
Exploitative leadership	−0.29	29.40
Interactional injustice	−0.648	31.00
Managing my boss	0.15	66.96

### 5.3 Necessary Condition Analysis

**FIGURE 4:**
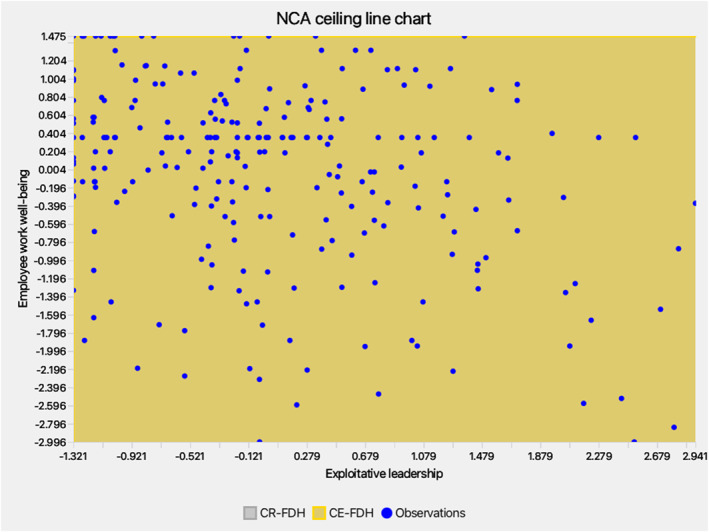
*NCA chart‐EL*.

**FIGURE 5:**
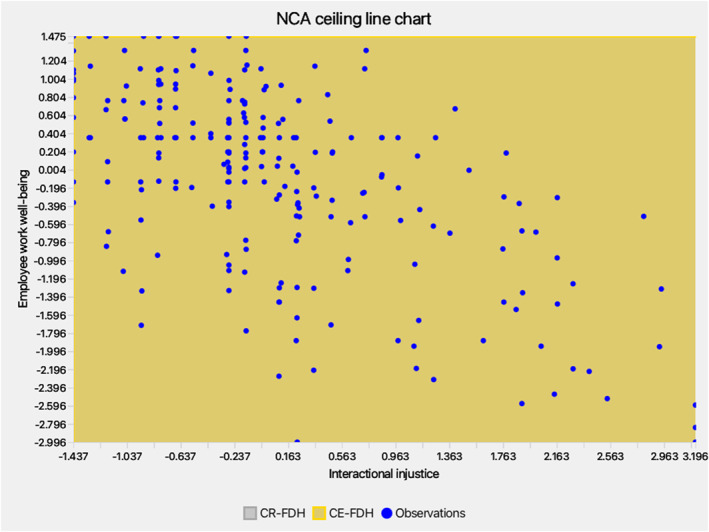
*NCA chart‐IIJ*.

**FIGURE 6:**
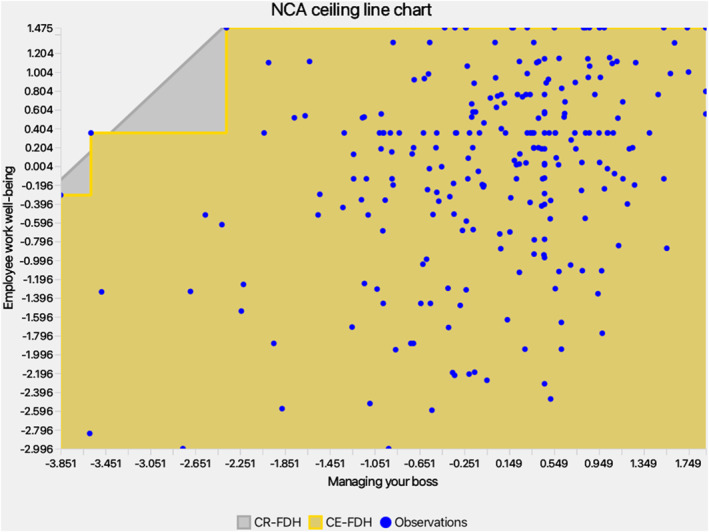
*NCA chart‐MYB*.

NCA complemented PLS‐SEM by identifying necessities (Dul 2016; Richter et al. 2020, 2023). Scatterplots (Figures 4, 5, 6) and effect sizes (Table 6) showed no significant necessities (CE‐FDH/CR‐FDH ≈ 0, *p* > 0.05; accuracy > 95%). Bottlenecks indicated MYB requires minimum levels (e.g., 3.042 for 90% WWB), but EL/IIJ were non‐necessary (NN), suggesting well‐being can occur without low EL/IIJ, per resource buffering (Bakker and Demerouti 2017).

**TABLE 6:** Bottleneck table—percentiles and NCA effect sizes.

Bottleneck CPB	WWB	EL	IIJ	MYB
0.00%	−2.996	NN	NN	NN
10.00%	−2.549	NN	NN	NN
20.00%	−2.102	NN	NN	NN
30.00%	−1.655	NN	NN	NN
40.00%	−1.208	NN	NN	NN
50.00%	−0.761	NN	NN	NN
60.00%	−0.314	NN	NN	NN
70.00%	0.134	NN	NN	0.760
80.00%	0.581	NN	NN	3.042
90.00%	1.028	NN	NN	3.042
100.00%	1.475	NN	NN	3.042
NCA effect sizes (accuracy and fit are 100%)				

**Construct**	**CPB CE‐FDH**	**Accuracy**	**Permutation *p*‐Value**	
EL	−0.00	100%	0.80	
IIJ	0.00	100%	1.00	
MYB	0.07	100%	0.20	

Abbreviations: EL = Exploitative leadership, IIJ = Interactional injustice, MYB = Managing your boss, NCA: Necessary condition analysis; NN = Not necessary; WWB = Employee workplace well‐being.

## 6 Discussion

This study advances our understanding of the detrimental effects of exploitative leadership (EL) on employee's WWB by elucidating the mediating role of IIJ and the moderating influence of MYB strategies. Grounded in the COR theory (Hobfoll et al. 2018) and the JD‐R model (Bakker and Demerouti 2017), with the TTS (Lazarus and Folkman 1984) as a supportive framework for coping processes, our findings from a two wave survey of 263 U.S. employees, analysed via PLS SEM, IPMA, and NCA, reveal a pathway: EL positively predicts IIJ, which in turn negatively affects WWB, fully mediating the EL WWB relationship. Notably, MYB buffers the EL IIJ link, weakening it at higher levels of MYB, and extends this moderation to the indirect pathway. IPMA highlights IIJ as a high importance negative predictor of WWB, while NCA indicates no necessary conditions but underscores MYBs role in enabling higher WWB levels. Consistent with Wang et al. (2024), who highlight moral potency's protective role, our results show MYB empowers agency to mitigate harm, offering practical strategies for well‐being enhancement.

These results align with CORs resource loss spirals, where EL depletes socioemotional resources like trust and dignity, manifesting as heightened IIJ perceptions (Halbesleben et al. 2014). From a JD‐R perspective, EL acts as a chronic job demand that strains interpersonal resources, eroding well‐being unless buffered by personal resources such as MYB (Bakker and Demerouti 2017). TTS complements this by framing MYB as a proactive coping mechanism that reframes EL as a manageable challenge, reducing injustice appraisals (Lazarus and Folkman 1984). Collectively, these findings integrate the theories into an overarching narrative: EL initiates depletion and threat appraisal, IIJ amplifies the strain on WWB, and MYB interrupts the cycle through resource conservation and reappraisal. Empirically, our mediation finding extends prior work linking destructive leadership to well‐being decrements through justice perceptions (e.g., Schmid et al. 2019), while the moderated mediation emphasises follower's agency in mitigating harm, echoing calls for follower centric views in leadership research (Krasikova et al. 2013). This shifts the narrative from passive victimization under destructive leadership to empowered coping, addressing who cares (organizations facing well‐being crises), so what (proactive strategies matter), and what we learn (integrated resource stress models enhance prediction).

The buffering effect of MYB is particularly intriguing, albeit modest, as it indicates that employees can proactively conserve resources and adapt their behaviors to counteract EL's manipulative tactics, such as taking credit or exerting pressure (Schmid et al. 2019). This aligns with evidence that proactive followership enhances resilience in adverse leadership contexts (Gajendran et al. 2022), though it also raises questions about the sustainability of such strategies, given proactive potential for resource depletion (Lin and Johnson 2015). Overall, these insights contribute to theory by rebutting overly deterministic views of destructive leadership (e.g., EL inevitably erodes well‐being) and supporting integrated models where follower actions moderate outcomes, relating to existing findings on buffering in high demand environments (Xanthopoulou et al. 2009).

### 6.1 Theoretical Implications

Our study contributes to the destructive leadership domain by integrating and extending COR and JD‐R, with TTS supporting coping dynamics, particularly for the nascent EL construct through a narrative lens of theory building (Shepherd and Suddaby 2017). This enriches ELs nomological network by demonstrating its indirect erosion of WWB via IIJ, illuminating how self‐interested behaviors (e.g., manipulation, egoism) trigger resource depletion spirals under COR, leading to resource strains and diminished well‐being (Hobfoll et al. 2018). By positioning IIJ as a full mediator, we address gaps in destructive leadership research, which has often overlooked perceptual mechanisms like injustice in favor of direct effects (A. Bajaba et al. 2023; S. Bajaba et al. 2025; Syed et al. 2021).

Second, introducing MYB as a moderator advances JD‐R by conceptualising it as a personal resource that attenuates the demand strain link in high EL contexts (Bakker and Demerouti 2017; Xanthopoulou et al. 2009). Unlike prior studies focussing on organizational buffers (e.g., social support; Wu et al. 2021), our moderated mediation model highlights follower‐initiated coping, where TTS adds unique value beyond JD‐R's resource focus by emphasising the sequential appraisal process, primary evaluation of EL as a threat and secondary reappraisal via MYB strategies, that enables adaptive responses (Lazarus and Folkman 1984; Shepherd and Suddaby 2017). This contributes to proactivity literature by delineating MYBs conditional benefits, stronger in mitigating IIJ under EL, while cautioning against over reliance, as proactivity can lead to exhaustion if misaligned with situational demands (Parker et al. 2019; Strauss et al. 2017). We extend Gajendran et al. (2022) by linking MYB to justice outcomes, suggesting it fosters adaptive alignment that preserves relational resources.

Third, the complementary use of IPMA and NCA provides methodological innovation, revealing MYBs high performance in enabling WWB without being necessary, consistent with JD‐Rs sufficiency logic (Ringle and Sarstedt 2016; Richter et al. 2020). This multi method approach addresses critiques of PLS SEMs focus on average effects by identifying practical priorities (e.g., reducing IIJ) and bottlenecks, enhancing theoretical precision in resource‐based models (Hauff et al. 2024). Collectively, these insights counterbalance the fields emphasis on positive leadership (e.g., transformational; Fuller et al. 2022) by underscoring destructive forms pervasive impacts, often rooted in power imbalances (Cortina et al. 2001; Tepper 2007), and advocate for integrated theories that incorporate follower agency.

### 6.2 Practical Implications

Our findings offer actionable guidance for organizations aiming to mitigate ELs harms and bolster WWB. Given ELs positive association with IIJ and indirect toll on well‐being, leaders should prioritise ethical development programs that kerb self‐interested traits like narcissism or Machiavellianism during selection and promotion (S. Bajaba et al. 2022). For instance, incorporating 360‐degree feedback and training on interdependence can foster awareness of exploitative tendencies, reducing their prevalence (Schmid et al. 2019).

The mediating role of IIJ underscores the need for justice enhancing policies, such as transparent communication protocols and equity audits, to rebuild trust and prevent conflict escalation (Colquitt et al. 2001). Organizations could implement employee assistance programs (EAPs) providing psychological support to replenish depleted resources, aligning with COR principles (Hobfoll et al. 2018). Moreover, given MYBs buffering effect, training initiatives should equip employees with proactive skills, understanding boss priorities and adapting communication, in high ambiguity or low routinisation roles (Gajendran et al. 2022). For instance, organizations could integrate MYB training into leadership development programs, teaching employees to align with boss priorities through workshops, as suggested by Gajendran et al. (2022), to enhance resilience in EL contexts. This empowers subordinates to navigate EL without overexertion, potentially through workshops on strategic coping (Parker et al. 2019).

Broader structural changes are essential: cultivating cultures of transparency via regular feedback, whistleblower protections, and performance metrics emphasising fairness can deter exploitative behaviors (Tepper et al. 2017). By investing in these, organizations not only safeguard well‐being but also can enhance retention and productivity, countering ELs hidden costs.

### 6.3 Limitations and Future Directions

Despite its strengths, this study has limitations that suggest avenues for refinement. The two‐wave design with a 2‐week lag mitigates common method bias but does not fully establish causality; future research could adopt longer longitudinal or experimental designs, incorporating multi‐source data (e.g., leader ratings) to validate temporal sequences (Maxwell and Cole 2007; Podsakoff et al. 2012). Self‐reported measures, while appropriate for perceptual constructs, may introduce subjectivity; we acknowledge potential pitfalls associated with self‐reports, including biases that may inflate associations due to common method variance (Podsakoff et al. 2003). To address this, we implemented procedural remedies (e.g., anonymity, counterbalancing items) and statistical tests (e.g., Harman's single‐factor test; partial correlation with marker variable); however, the interactions found in the current study would be difficult to detect if common method variance was present (Siemsen et al. 2010). Future studies should explore multi‐rater approaches to further reduce these risks.

Additionally, the sample's demographic skew, 60% male and a mid‐career focus (45% aged 30–39 years), may limit generalisability, potentially influencing perceptions of injustice or coping strategies differently across gender or career stages. This includes the vast majority (nearly 90%) having over 5 years of overall work experience, which restricts insights into whether less experienced employees (e.g., under 3 years) perceive and respond to EL differently, such as with altered injustice appraisals or MYB efficacy. Future studies should test whether demographics moderate these relationships, including by using samples with balanced work experience distributions to examine career‐stage variations, using diverse samples to enhance external validity.

The U.S.‐centric sample limits generalisability: cross‐cultural replications are needed, as EL perceptions may vary by collectivism or power distance (Vogel et al. 2015). Focussing solely on IIJ overlooks other justice facets (e.g., distributive, procedural); exploring their interplay could provide a holistic view (Colquitt 2001). Additionally, while MYB buffered effects, its long‐term sustainability warrants investigation, particularly in depleting contexts (Lin and Johnson 2015). The high EL‐IIJ correlation (*r* = 0.56) suggests potential conceptual overlap, though analyses confirm unique variance; multi‐source data could further disentangle this. We also note the moderation effect's marginal significance (*p* = 0.047) reflects a small predictive power, potentially due to sample size or contextual factors, and warrant replication in diverse settings, though such effects align with norms in moderation research and contribute meaningfully to understanding employee agency (Aguinis et al. 2017; Dawson 2014).

Future studies could examine alternative mediators like psychological contract breach, where EL's unreciprocated demands erode vitality and extra‐role behaviors (Gakovic and Tetrick 2003), or employ experience sampling methods (ESM) to capture real‐time appraisals of EL stressors and MYB responses, addressing intra‐individual fluctuations overlooked in aggregated designs (Gabriel et al. 2019). Moderators such as personality types (e.g., proactive vs. adaptive; Fuller et al. 2018) or sectoral differences (public vs. private; Kearney et al. 2009) merit exploration, as do interventions testing MYB training's efficacy or alternative buffers like mindfulness practices, which enhance self‐regulation and reduce reactivity to destructive leadership (Glomb et al. 2011). Additionally, longitudinal research could investigate the long‐term implications of MYB behaviors under EL, such as potential erosion of follower self‐esteem over time, as employees may mask the leader's hindering qualities by convincing themselves of lesser harm or prioritising the leader's needs over their own (Lin and Johnson 2015; Parker et al. 2019). This could extend to broader outcomes like employee performance, innovativeness, and loyalty, uncovering both benefits and hidden costs of proactive upward management in exploitative contexts.

Future studies could also explore alternative theoretical lenses like SET to examine justice social comparisons and exchange norms in cross‐cultural EL contexts, potentially revealing how reciprocity violations moderate IIJ's effects beyond resource‐based models (Cropanzano et al. 2017; Koopman et al. 2020; Vogel et al. 2015; Wu et al. 2021).

## 7 Conclusion

This study illuminates how EL undermines WWB through IIJ, while MYB offers a proactive shield by weakening this pathway. Our findings affirm EL as a resource‐depleting demand that fosters injustice and stress yet highlight employees' capacity to adapt and thrive via strategic followership. These contributions extend destructive leadership literature, emphasise follower agency, and provide practical tools for fostering resilient workplaces.

## Author Contributions

All the authors contributed equally to this work.

## Funding

The authors have nothing to report.

## Ethics Statement

All procedures performed in studies involving human participants were in accordance with the ethical standards of the institutional and/or national research committee and with the 1964 Helsinki Declaration and its later amendments or comparable ethical standards.

## Consent

Informed consent was obtained from all individual participants included in the study.

## Conflicts of Interest

The authors declare no conflicts of interest.

## Supporting information


Supporting Information S1

## Data Availability

The datasets generated during and/or analyzed during the current study are available from the corresponding author upon reasonable request.
