# Supplementary material for: Safeguarding Well‐Being Under Exploitative Leaders: The Buffering Effects of Follower Strategies on Perceived Injustice
Source: Stress Health. 2025 Nov 28;41(6):e70127. doi: 10.1002/smi.70127 (PMC12661555; doi:10.1002/smi.70127)
Supplement: Supplementary file 1 — Supporting Information S1 [file SMI-41-e70127-s001.docx]

**Appendix A:** Measurement scales and sources

Note: All scales used 5-point Likert anchors (1=strongly disagree to 5=strongly agree).

| **Exploitative leadership:** | |
| --- | --- |
| **Measurement items** | **Source** |
| 1. Takes it for granted that my work can be used for his or her personal benefit. 2. Sees employees as a means to reach his or her personal goals. 3. Values the achievement of his or her own goals over the needs of the employees. 4. Puts me under pressure to reach his or her goals. 5. Increases my workload without considering my needs in order to reach his or her goals. 6. Does not consider my workload when new tasks need to be assigned. 7. Gives me tedious tasks if he or she can benefit from it 8. Does not give me opportunities to further develop myself professionally because his or her own goals have priority. 9. Gives me boring routine tasks when he or she can benefit from it. 10. Uses my work to get himself or herself noticed. 11. Passes the team's work off as his or her own. 12. Uses my work for his or her personal gain. 13. Plays my colleagues and me off against each other to reach his or her goals. 14. Manipulates others to reach his or her goals. 15. Does not hesitate to manipulate or deceive employees in order to reach his/her goals. | Schmid et al.'s (2019 |

| **Interactional injustice:** | |
| --- | --- |
| **Measurement items** | **Source** |
| 1. When decisions are made about my job, the general manager treats me with kindness and consideration. 2. When decisions are made about my job, the general manager treats me with respect and dignity. 3. When decisions are made about my job, the general manager is sensitive to my personal needs. 4. When decisions are made about my job, the general manager deals with me in a truthful manner 5. When decisions are made about my job, the general manager shows concern for my rights as an employee. 6. When decisions are made about my job, the general manager discusses the implications of the decisions with me. 7. When decisions are made about my job, the general manager offers adequate justification for these decisions. 8. When decisions are made about my job, the general manager offers explanations that make sense to me. 9. When decisions are made about my job, the general manager explains these decisions very well and in great detail. | Moorman (1991) |

| **Managing your boss:** | |
| --- | --- |
| **Measurement items** | **Adopted From** |
| 1. I proactively attempt to get a better understanding of my boss's priorities. 2. I prioritize my tasks to meet my boss's work goals. 3. I seek to better understand my boss's work goals. 4. I think about what is important to my boss when planning my work. 5. It's important to try to figure out my boss's top work priorities. 6. I attempt to see work-related matters from my boss's perspective. 7. I strive to understand my boss's preferred work styles. 8. I learn to adapt and resolve my boss's pressing issues. 9. I try to help my boss to achieve his/her work goals. 10. I regularly align my communication style with my boss's preferred communication style to meet my boss's communication preferences. | Gajendran et al. (2022) |

| **Employee workplace well-being:** | |
| --- | --- |
| **Measurement items** | **Source** |
| 1. I am satisfied with my work responsibilities. 2. In general, I feel fairly satisfied with my present job. 3. I find real enjoyment in my work. 4. I can always find ways to enrich my work. 5. Work is a meaningful experience for me. 6. I feel basically satisfied with my work achievements in my current job. | Zheng et al. (2015 |

| **Attitude toward color blue:** | |
| --- | --- |
| **Measurement items** | **Source** |
| 1. Blue is a beautiful color. | Miller & Simmering (2022) |
| 1. Blue is a lovely color. |  |
| 1. Blue is a pleasant color. |  |
| 1. The color blue is wonderful |  |
| 1. Blue is a nice color. |  |
| 1. I think blue is a pretty color |  |
| 1. I like the color blue. |  |

| **Demographic** | |
| --- | --- |
| Age | - 19 to 29 - 30 to 39 - 40 to 49 - 50 to 59 - 60 and more |
| Gender | - Male   Female |
| 1. Education | - High school diploma/A-levels - Technical/community college - Undergraduate degree (BA/BSc/other) - Graduate degree (MA/MSc/MPhil/other) - Doctorate degree (PhD/other) |
| 1. Working experience with the current organization | - 7-12 months - 1-2 years - 2-5 years - More than 5 years |
| 1. Working experience with the current leader | - Less than 6 months - More than 6 months and less than 18 months - Over 18 months and less than 3 years - 3 years and less than 5 years - Over 5 years |
| 1. Working experience overall | - Less than 6 months - More than 6 months and less than 18 months - Over 18 months and less than 3 years - 3 years and less than 5 years - Over 5 years |
